# Time to Reach Full Enteral Feeding and Its Predictors among Very Low Birth Weight Neonates Admitted in the Neonatal Intensive Care Unit: A Follow-Up Cohort Study

**DOI:** 10.1155/2024/9384734

**Published:** 2024-06-24

**Authors:** Belay Alemayehu Getahun, Sileshi Mulatu, Hailemariam Mekonnen Workie

**Affiliations:** ^1^ Felege Hiwot Comprehensive Specialized Hospital, Bahir Dar, Ethiopia; ^2^ Bahir Dar University College of Medical and Health Sciences Department of PCHN, Bahir Dar, Ethiopia

## Abstract

**Background:**

Feeding is crucial for very low birth weight neonates to grow and develop properly. This study aims to determine the time to achieve full enteral feeding and predictors among neonates admitted at Felege Hiwot Comprehensive Specialized Hospital.

**Methods:**

An institutional retrospective follow-up study design was conducted among 332 very low birth weight neonates from July 1, 2018, to June 30, 2021. Samples were selected through a computer-generated simple random sampling method, and the data were entered into Epi data version 4.6 and then exported to STATA version 16 for analysis. Kaplan–Meier with the log-rank test was used to test for the presence of difference in survival among predictor variables. Model goodness of fit and assumptions were checked by the Cox–Snell residual and the global test, respectively. Variables with *p* value <0.25 in the bi-variable analysis were fitted to the multivariable Cox-proportional hazard model. Finally, the adjusted hazard ratio (AHR) with 95% CI was computed, and variables with a *p* value less than 0.05 in the multivariable Cox regression analysis were considered significant predictors of time to reach full enteral feeding.

**Results:**

A total of 332 neonates were followed for 2,132 person days of risk time and 167 (50.3%) of very low birth weight neonates started full enteral feeding. The overall incidence rate of full enteral feeding was 7.8 per 100 person day observations. The median survival time was 7 days. Very low birth weight neonates delivered from pregnancy-induced hypertension-free mothers (AHR: 2.1; 95% CI: 1.12, 3.94), gestational age of ≥33 weeks (AHR: 5,; 95% CI: 2.29, 11.13), kangaroo mother care initiated (AHR: 1.4; 95% CI: 1.01, 2.00), avoiding prefeed residual aspiration (AHR: 1.42; 95% CI: 1.002–2.03), and early enteral feeding (AHR: 1.5; 95% CI: 1.03, 2.35) were significant predictors of full enteral feeding.

**Conclusions:**

According to this study, the time to achieve full enteral feeding was relatively short. Therefore, healthcare professionals should emphasize achieving full enteral feeding and address hindering factors to save the lives of VLBW neonates.

## 1. Background

The World Health Organization (WHO) has defined very low birth weight neonates (VLBW) as weighing between 1,000 g and 1,500 g [1]. Nutrient requirements increase for VLBW newborns due to low nutrient reserves and vulnerability to stressors. The postnatal growth rate is recommended to be equivalent to intact fetuses at the same gestational age [2].

Enteral feeding is crucial for very low birth weight (VLBW) infants' growth and health. It is important to avoid total parenteral nutrition (TPN) and complete enteral feeding quickly to maintain proper nutrition and growth while preventing negative effects [3, 4].

Time to full enteral feeding (TFEF) is when neonates start receiving all their prescribed nutrition as milk feeds. Full enteral feeding (FEF) means infants get 120 to 150 mL/kg/day of all their prescribed nutrition as milk feeds (either human milk or formula). Breast milk is better for VLBW babies due to its health benefits, but if not available, preterm formula or donor human milk can be used in NICUs [4–9].

The outcomes in very low birth weight newborns, such as nutrient intake, the risk of necrotizing enterocolitis, time to full enteral feeding, and growth and development, are influenced by early enteral feeding methods, especially the timing of introduction and the rate of progression of milk feeds. In addition, early enteral feeding is preferable to parenteral as it has biological effects with serious implications for later health and is safer and easier [10–12].

The common approach for feeding very low birth weight (VLBW) neonates is to start with low-volume enteral milk feeds and gradually increase the volume over 1–2 weeks. Conservative enteral feeding regimens delay gastrointestinal hormone secretion and motility, diminish the functional adaptation of the gastrointestinal tract, and disrupt the patterns of microbial colonization. Intestinal dysmotility and dysbiosis might exacerbate feed intolerance and delay the establishment of enteral feeding independently of parenteral nutrition [4, 13].

It is important to prioritize early introduction and rapid achievement of full enteral feeding (FEF) for VLBW infants to reduce the need for central venous catheters, risk of infection, liver problems, and length of hospital stay. Delaying the start of enteral feeding could result in adverse outcomes. Intermittent feeding boosts weight gain and stomach emptying rate, while continuous feeding is effective in the transition to full enteral nutrition and in reducing the risk of hypoxic-ischemic intestinal injury. However, studies report no difference between intermittent and continuous feeding in terms of transition times to full enteral feeding. It is crucial to avoid keeping newborns on nothing by mouth (NPO) for the first several days, as it raises the risk of death and the onset of hypoglycemia [13–20].

The requirement for FEF frequently competes with the physiological immaturity of VLBW neonates' gastrointestinal function as well as the onset of different comorbidities during the neonatal period. There is evidence that the timing of introduction and pace of advancement of breastfeeds, in particular, have an impact on vital newborn VLBW outcomes, such as nutrient intake, the risk of necrotizing enterocolitis (NEC), growth, and development. Another study revealed that sluggish feeding is more likely to cause NEC than early introduction and steady advancement of enteral feeding [4, 12, 21–23].

Even though delayed full enteral feeding in very low birth weight neonates has potential disadvantages, studies on the time to reach full enteral feeding and predictors are limited. Early full enteral feeding results in earlier removal of vascular catheters, less sepsis, and other catheter-related complications in VLBW neonates. Delayed full enteral feeding and prolonged duration of parenteral nutrition are associated with infections and metabolic complications, increasing short-term and long-term morbidity and mortality, prolonged hospital stay, increased risk of adverse outcomes, and affecting growth and development [10, 24, 25].

To prevent complications related to full enteral feeding in VLBW neonates, it is important to determine the time to reach full enteral feeding and identify predictors in the NICU.

## 2. Methods

### 2.1. Study Area and Period

This study was conducted from May 13, 2022, to June 12, 2022, in Bahir Dar City at Felege Hiwot Comprehensive Specialized Hospital. Bahir Dar is the capital city of Amhara regional state located in northwest Ethiopia, 565 km away from Addis Ababa, the capital city of Ethiopia. Felege Hiwot Comprehensive Specialized Hospital is the former hospital, which was established in 1963 as a referral hospital. Now, it has 410 beds and serves more than 5 million people. It is organized into different wards, namely, medical ward, surgical ward, gynecology and obstetrics ward, orthopedics ward, oncology ward, pediatric ward, adult ICU, NICU, and different outpatient departments. According to the information from the NICU coordinator, the ward has 71 neonatal beds with an average annual admission of 2,099 neonates, and currently, the total number of nurses, general practitioners, and pediatric physicians working there are 35, 8, and 3, respectively.

### 2.2. Study Populations and Their Characteristics

An institution-based retrospective cohort study was conducted among VLBW neonates admitted at FHCSH from July 1, 2018, to June 30, 2021. Those neonates with a body weight of 1,000 to 1,500 g and admitted to the NICU of FHCSH within 24 hours of birth from July 1, 2018, to June 30, 2021, were included in this study, and those neonates who had gastroschisis, omphalocele, were transferred to another hospital or died within 24 hours of birth, and with incomplete charts (if important variables were missing such as mode of delivery, starting time of enteral feeding, type of feeding, frequency of feeding, daily feeding volume advancement not included) were excluded from this study. Each VLBW neonate's chart was selected through a simple random sampling method used as a study unit.

### 2.3. Sample Size Determination

The sample size was determined using the double population proportion difference formula by using predictor variable time to the initiation of enteral feeding from another study conducted in China. Time to the initiation of enteral feeding was considered a statistically significant independent predictor of time to reach full enteral feeding [26]. The sample size needed for this study was calculated using STATA version 16, considering the following statistical assumptions: two-sided significant level (*α*) of 5%, power 80%, Za/2 = *Z* value at 95% confidence interval = 1.96, hazard ratio (HR) = 1.52, the survival probability of event = 0.59, the proportion of withdrawal = 10% incomplete charts with a one-to-one allocation ratio of exposed to nonexposed was assumed. Finally, the total sample size was 338.

### 2.4. Sampling Technique and Procedures

First, all VLBW neonates' card numbers were obtained from the NICU registration logbook. The total number of VLBW neonates who were admitted from July 1, 2018, to June 30, 2021, was 1,350. All VLBW neonatal medical registration numbers were listed with a sample frame from 1 to 1,350. The study units from the sampling frame were selected by a simple random sampling technique through a computer-generated system using a statistical package for social sciences software version 25. Finally, a total of 338 charts were selected.

### 2.5. Data Collection Tools and Procedures

Data were extracted from patient charts by using structured data collection tools adapted from previous studies as chart review checklists were included by reviewing different related literature in terms of prenatal, neonatal, and enteral feeding variables [27–32]. The VLBW neonates' medical registration numbers were first obtained from the NICU ward Federal Ministry of Health (FMOH) registration logbook. After that, the required numbers of medical registration charts were selected using a sampling procedure. The selected medical cards were obtained from the medical record office. The data were collected from admitted neonates within 24 hours after birth to an event or censored occurred within the follow-up period. Two BSc nurses were recruited for data collection, and one MSc nurse was assigned as a supervisor.

### 2.6. Data Quality Control

The data extraction checklist was adapted and structured from literature and commented on by senior pediatricians for its consistency and completeness. A 1-day training was given to data collectors and supervisors before data collection. A pretest was done on 5% of VLBW neonates' cards before data collection. Close supervision was carried out by the supervisor and the PI during data collection time. Finally, all the collected data were checked by the investigator for completeness and consistency, and everyday data cleaning was done. Once the data were extracted from patient charts, they were coded to avoid duplication.

### 2.7. Data Processing and Analysis

The data were cleaned and coded by using Epi data version 4.6. The consistency of data was also checked before analysis and exported to STATA version 16 statistical software. Descriptive statistics (mean with standard deviation for normal distributions, median with the interquartile for skewed data, and frequency with percentages) were computed depending on the nature of the variables. The results were presented using texts, charts, graphs, and tables. The outcome of each participant was dichotomized into censored and event. The incidence density rate (IDR) was calculated for the entire study period. Kaplan–Meier (KM) was used to estimate the median survival time and cumulative probability of survival, and a KM plot with a log-rank test was used to compare survival curves. Before performing the Cox-proportional hazard regression, the model goodness of fit was checked by Cox–Snell residuals, and assumptions were checked by using the Schoenfeld residual test. Those variables with a *p* value > 0.05 were entered into the model. Multicollinearity was also checked. For each independent predictor, bivariable Cox proportional hazard regression was performed. Then, the variables with *p* value < 0.25 were included in the multivariable Cox proportional hazard regression. Adjusted hazard ratio with a 95% confidence interval and *p* value < 0.05 was used to measure the strength of association and considered as a statistical significance predictor of time to reach full enteral feeding.

### 2.8. Operational Definitions

  Very low birth weight neonate: neonate's birth weight is between 1,000 g and 1,500 g [33]
  Full enteral feeding: an infant receiving 120 to 150 mL/kg/d of either preterm formula or maternal breast milk sustained for 24 h and does not receive any supplemental parenteral fluids or nutrition [5, 34]
  Time to reach full enteral feeding: this is the time when neonates start full enteral feeding up to 7 days of age after birth [5, 35]
  Early enteral feeding: introduction of enteral feeding at birth up to 3 days of age [36]
  Late enteral feeding: introduction of enteral feeding after 3 days of birth [36, 37]
  Slow advancement of enteral feeding: increments of enteral feeding by 15–20 mL/kg/day [35]
  Faster advancement of enteral feeding: increments of enteral feeding by 30–40 mL/kg/day [35]
  Survival status: outcome of VLBW neonate, either event or censored
  Event: all VLBW neonates with the outcome of FEF
  Censored: all VLBW neonates with predictors other than an event (lost to follow-up, died after 24 hours of birth, not FEF over the follow-up period, referred to another health facility before FEF, and against medical treatment before FEF)
  Survival time: it is the time from admission within 24 hours of birth to NICU up to the occurrence of an event/FEF
  Follow-up time: from the time of admission within 24 hours of birth until either an event or censorship occurs within 7 days

## 3. Results

### 3.1. Prenatal Information

Among VLBW neonates admitted to the NICU of FHCSH from July 1, 2018, to June 12, 2021, a total of 338 charts were reviewed. Of these, 332 medical records were included in the analysis which provided a completeness rate of 98%. From the reviewed charts, a majority (93.3%) of VLBW neonates were delivered from chorioamnionitis-free mothers. Among these, 51.6% could start full enteral feeding within seven days. Nearly one-fourth (22%) of very low birth weight neonates were born from mothers treated with corticosteroid prophylaxis. Of them, more than half (53.4%) started full enteral feeding within seven days of age. Among VLBW neonates delivered from PIH-free mothers, 58.2% achieved full enteral feeding (Table 1).

### 3.2. Neonatal Information

The majority (69.9%) of VLBW neonates were ≥33 weeks of gestational age. Among them, greater than two-thirds (69.3%) could start full enteral feeding. The median weight and median GA of VLBW neonates were 1,430 (IQR: 1,350–1,480 g) and 33 (IQR: 32-34 weeks), respectively. More than half (56.33%) of the total observations were delivered through CS. Regarding KMC initiation, greater than one-third (40.36%) were initiated on KMC (Table 2).

### 3.3. Enteral Feeding Information

More than half (62.05%) of the participants started enteral feeding before 3 days of age and nearly two-thirds (61.75%) were on breast milk. Nearly half (43.98%) of the neonates were checked for prefeed residual aspiration, and more than one-third (42.47%) were fed every 6 h per day (Table 3).

### 3.4. Survival Status of Neonates on Time to Reach FEF

**Figure 1:**
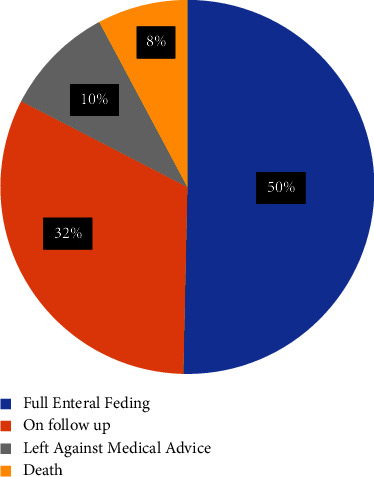
Outcome status of time to reach FEF among VLBW neonates admitted at FHCSH, Bahir Dar City, northwest Ethiopia, from 2018 to 2021.

Three hundred thirty-three study participants were followed for a total of 2,132 person days' risk time, with a minimum of 2 days and a maximum of 7 days observation. The mean follow-up time was 6.4 days and the median follow-up time was 7 with an IQR of 6–7 days. During the follow-up time, 167 (50.3%) neonates were fully enteral fed. Of the total study participants, 107 (32.2%) were on follow-up at the end of the study period, 32 (9.6%) were left against medical advice, and 26 (7.8%) died (Figure 1) The cumulative incidence probability of starting full enteral feeding was 50.3%; among this, by the end of 3, 4, 5, 6, and 7 days, it was 0.3, 1.2, 3.6, 8, and 38.4, respectively. The overall incidence density rate (IDR) of full enteral feeding was 7.8 per 100 (95% CI: 7) person days. The incidence rate that VLBW neonates start FEF was 0.3, 3.8, 9.9, and 53.2 per 100 person days in the first 4, 5, 6, and 7 days after birth, respectively. The median survival time to reach full enteral feeding was 7 (95% CI: 7) days.

### 3.5. Survival Probabilities of FEF among VLBW Neonates

**Figure 2:**
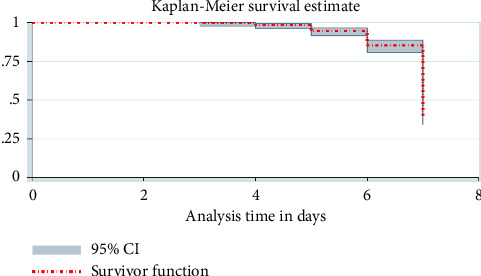
The Kaplan–Meier survival estimates of time to reach FEF among VLBW neonates admitted at FHCSH, Bahir Dar City, northwest Ethiopia, from 2018 to 2021 (*N* = 332).

The estimated cumulative survival probability of not full enteral feeding was 99.69% (95% CI: 0.9785-–0.9996) for the first 3 days, 98.44% (95% CI: 0.963–0.9935) at the end of the fourth day, 94.47% (95% CI: 0.9126–0.9653) at the end of the fifth day, 84.87% (95% CI: 0.8019–0.8852) at the end of the sixth day, and 25.89% (95% CI: 0.2021–0.3192) at the end of the seventh day of the follow-up period correspondingly. The finding illustrates that the overall full enteral feeding probability of very low birth weight neonates admitted at FHCSH was increasing as follow-up time increased where the highest incidence rate of time to reach full enteral feeding happened during the seventh day (Table 4 and Figure 2).

### 3.6. Log-Rank Test Result

**Figure 3:**
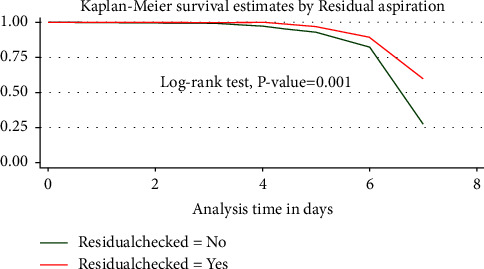
Kaplan–Meier survival estimate of time to reach full enteral feeding based on the prefeed residual aspiration of VLBW neonates admitted at FHCSH, Bahir Dar City, northwest Ethiopia, from 2018 to 2021 (*N* = 332).

In addition to the overall survival estimate, to assess the difference between groups, the survival experience of neonates with various categorical variables was computed. A log-rank test was used to determine whether the difference in FEF's survival experience was statistically significant (*p* 0.05) (Figure 3).

### 3.7. Cox-Proportional Hazard Assumptions

Each variable underwent the scaled Schoenfeld residuals proportional hazard assumption test, and the overall test was done. The *p* value was >0.05 for each variable as well as the overall global test (*p* value = 0.6875). This indicates that we fail to reject the null hypothesis; it assures that the assumption is satisfied (Table 5).

### 3.8. Model Goodness-of-Fitness Test

**Figure 4:**
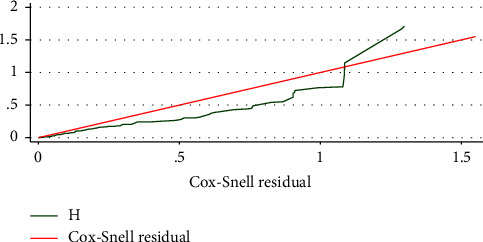
Cox–Snell residual test to the goodness of fit of Cox proportional hazard model among very low birth weight neonates admitted at FHCSH, Bahir Dar City, northwest Ethiopia, from 2018 to 2021 (*N* = 332).

The Cox–Snell residual test was employed to test the goodness of fit for the Cox proportional hazard regression model. The Cox–Snell residuals were estimated based on the Kaplan–Meier estimated survivor function. As we can see from the graph in Figure 4, the model closely follows the 45-degree line in this graphical display of the cumulative hazard versus Cox–Snell residuals curve.

### 3.9. Predictors of Time to Reach FEF

During bivariable Cox proportional regression analysis, five variables were found to be significant predictors of time to reach full enteral feeding. These five variables were gestational age, pregnancy-induced hypertension, initiation of KMC, avoiding prefeed residual aspiration, and early enteral feeding or early trophic feeding initiation. Furthermore, other variables that have a *p* value of < 0.25 in the bivariable analysis, including SGA, CPAP, and preeclampsia, were fitted into the multivariable Cox proportional hazard model. Then, the final multivariable Cox proportional regression model identified the following variables as statistical predictors of time to reach full enteral feeding. Pregnancy-induced hypertension, GA, the starting time of enteral feeding, KMC practice, and prefeed residual aspiration were statistically significant variables at 5% of the level of significance.

Very low birth weight neonates who were not prefeed residually aspirated were 1.4 times more likely to full enteral feeding as compared with neonates who were prefeed residually aspirated (AHR: 1.42; 95% CI: 1–2.03). Neonates with a gestational age of greater than or equal to 33 weeks were five times more likely to have full enteral feeding as compared with a gestational age of 28–32 weeks (AHR: 5; 95% CI: 2.29, 11.13). Furthermore, VLBW neonates who initiated KMC were 1.4 times more likely to be fully enterally fed than those who did not initiate KMC (AHR: 1.4; 95% CI: 1.01, 2.00). In this regard, the hazard of FEF among neonates who have early enteral feeding was 1.56 times more likely as compared with neonates with late enteral feeding (AHR: 1.5; 95% CI: 1.03, 2.35). VLBW neonates delivered from PIH-free mothers were 2.1 times more likely FEF as compared with VLBW neonates delivered from mothers with PIH (AHR: 2.1; 95% CI: 1.12, 3.94) (Table 6).

## 4. Discussion

This study aimed to assess the time to reach FEF and its predictors among VLBW neonates admitted to the study hospital. In this study, the incidence of FEF was 7.8 per 100 person days of risk time. At the end of follow-up, 50.3% (95% CI: 44.9%, 55.7%) of very low birth weight neonates were fully enteral fed. Among those who started FEF, only one (0.3%) of the VLBW neonates started within the first 3 days of birth. In this study, 0.3%, 1.2%, 3.6%, 8%, and 37% of very low birth weight neonates started FEF on 1, 4, 5, 6, and 7 days of birth, respectively. This indicates that only a small proportion of VLBW neonates started FEF within the first 7 days of birth. This finding is lower as compared to institution-based retrospective cohort studies conducted in Ethiopia (63.4%) [38], Italy (95.2%) [29], and the United States of America (South Carolina) (83%) [35]. The median survival time of full enteral feeding among VLBW neonates in this study was 7 days. The median survival time to reach FEF in this study was shorter when compared with a study conducted in public hospitals in Hawassa City with a median survival time of 8 days (IQR: 7–10) [38]. This finding is in line with a study conducted in Kenya and Nigeria with a median of 8 (IQR 6-12) days [39], and Italy with a median time to reach FEF being 13 days (IQR 7–24 days) [29]. On the other hand, it indicates reaching full enteral feeding earlier as compared with a cohort study done in South China (IQR: 8–11 days) [27], a retrospective cohort study in the University of Alabama at Birmingham Hospital with a median of 11 days (IQR: 8-13) [28], in Indonesia with a median of 11 days (IQR: 8–21) [31], and a study in India with a median of 11 days (IQR: 8–15) [40]. The difference might be due to the study population differences, study setup, study design (retrospective versus prospective), study period, follow-up time, sample size difference, sociodemographic variations, and differences in regional variation in neonatal management protocols [29, 38].

According to this study, gestational age > 32 weeks, pregnancy-induced hypertension, early starting of enteral feeding or trophic feeding, prefeed residual aspiration, and initiation of KMC were statistically significant predictors of time to reach full enteral feeding among very low birth weight neonates.

The hazard of full enteral feeding among very low birth weight neonates born with greater than 32 weeks of gestation was five times more likely as compared to those born ≤ 32 weeks of gestation. This might be due to differences in physiological maturity among these groups of neonates in whom necrotizing enterocolitis and feeding intolerance are less common while gestational age increases. The finding is supported by a study done on tertiary hospital NICUs in different countries that revealed the higher gestational age was the reason for full enteral feeding [4]. Furthermore, a study done in Israel showed that as gestational age increased the time taken to full enteral feeding decreased [32].

Early enteral feeding was another statistically significant predictor. The hazard of starting full enteral feeding among very low birth weight neonates with early enteral feeding was 1.5 times more likely as compared to neonates with late enteral feeding. This finding might be because of accelerated gastrointestinal, physiological, endocrine, and metabolic maturity which allows infants to transition to full enteral feeding independent of parenteral nutrition more quickly [41]. This finding is also in line with a study that revealed that early enteral or trophic feeding stimulates gastrointestinal hormone secretion and motility, decreasing the time to reach full enteral feeding. In contrast, late enteral feeding may diminish the functional adaptation of the gastrointestinal tract and disrupt the patterns of microbial colonization [41].

Likewise, in this study, VLBW neonates who initiated KMC increased the hazard of full enteral feeding by 1.4 times as compared to their counterparts. This finding is supported by an observational study in India, which reported that kangaroo position during KMC reduces gastric residual volume, thereby improving feeding tolerance, and this could explain the shortened time to reach full enteral feeding [30]. Another study conducted in Bangladesh revealed that kangaroo mother care reduced the time to reach full enteral feeding due to early initiation of breastfeeding and increased mother to newborn bonding [42]. In addition, a guideline on very low birth weight neonates recommended that kangaroo mother care reduces the time to reach full enteral feeding [43].

Very low birth weight neonates who were delivered from pregnancy-induced hypertension-free mothers were 2.1 times more likely to commence full enteral feeding as compared with those very low birth weight neonates who were delivered from pregnancy-induced hypertense mothers. This finding was supported by a study conducted in northeastern Italy, which revealed that maternal hypertension delayed the time to reach FEF by 11.2%, probably because of decreased uteroplacental blood perfusion, leading to small size for gestational age, and also the difficulty in providing timely and effective care to the newborn [29]. On the other hand, very low birth weight neonates born from mothers diagnosed with maternal hypertension most likely developed NEC. As a result of this, the time to reach full enteral feeding was prolonged. This is supported by a study conducted in Israel that showed that maternal hypertension was an independent risk factor for the development of NEC in neonates of very low birth weight [44].

Likewise, the hazard of time to reach full enteral feeding among very low birth weight neonates who were not frequently prefeed residually aspirated was 1.42 times more likely as compared with their counterparts. This finding was supported by a study in Israel that suggested that avoiding routine gastric residual volume evaluations contributed to earlier attainment of full enteral feeding [32]. In addition, a study conducted in Italy showed that avoidance of routine prefeed evaluation of gastric residuals was associated with earlier starting of full enteral feeding, shortened duration of hospitalization, and also lower incidence of late onset sepsis [45]. These results were explained by the inappropriate discontinuation of enteral feeding with subsequent delays in the advancement of enteral nutrition associated with the routine prefeed assessment of gastric residuals [46]. Furthermore, a random control trial study at the University of Florida, USA, revealed that very low birth weight (VLBW) infants found that undergoing routine aspiration and evaluation of gastric residual aspiration delayed time to reach full feeding (150 mL/kg/d) by 6 days [47].

### 4.1. Limitations of the Study

Since the data were collected from a secondary source of medical records, other important predictors of time to reach full enteral feedings like availability of feeding milk and maternal and paternal sociodemographic status were not assessed. Study design and follow-up time also might affect the strengthening of this study. Therefore, we strongly recommended the next researchers to conduct a prospective cohort.

## 5. Conclusions and Recommendations

The overall median survival time to reach full enteral feeding was relatively short in the study hospital, with a significant proportion of VLBW neonates achieving full enteral feeding by the seventh day of age. Our analysis identified several predictors that influenced the time to reach full enteral feeding. Specifically, gestational age ≥ 33 weeks, early trophic or enteral feeding initiation, consistent kangaroo mother care practices, prevention of prefeed residual aspiration, and neonates born to mothers without pregnancy-induced hypertension were factors associated with delayed attainment of full enteral feeding. Therefore, tailored interventions focusing on optimizing feeding strategies for preterm neonates with lower gestational ages, promoting early and consistent kangaroo mother care, implementing protocols to prevent prefeed residual aspiration, and providing specialized care for neonates born to mothers with pregnancy-induced hypertension are recommended to improve the timeliness of achieving full enteral feeding in VLBW neonates.

## Figures and Tables

**Table 1:** Prenatal information of time to reach full enteral feeding among very low birth weight neonates admitted at FHCSH, Bahir Dar City, northwest Ethiopia, from 2018 to 2021.

Variables	Category	Outcome status				Total (332)	%	IDR/100/PDO
		FEF	%	Censored	%			
ANC corticosteroid Prophylaxis	No	128	49.4	131	50.57	259	78.0	7.7
	Yes	39	53.4	34	46.6	73	22.0	8.2

Preeclampsia	No	157	52.5	142	47.5	299	90.06	8.1
	Yes	10	30.3	23	69.7	33	9.94	4.7

Chorioamnionitis	No	160	51.6	150	48.4	310	93.3	7.9
	Yes	7	31.8	15	68.2	22	6.63	5.5

PIH	No	156	58.2	112	41.8	268	80.7	9.0
	Yes	11	17.2	53	82.8	64	19.3	2.7

PROM	No	145	52.3	132	47.7	277	83.43	8.1
	Yes	22	40	33	60	55	16.57	6.1

*Note.* PPROM = preterm prolonged rupture of membrane; PIH = pregnancy-induced hypertension, PDO = person-day observation.

**Table 2:** Neonatal and clinical information on time to reach FEF among VLBW neonates in the NICU of FHCSH. Bahir Dar City, northwest Ethiopia, from 2018 to 2021 (*N* = 332).

Variables		Outcome status				Total		IDR/100/PDO
		FEF	%	Censored	%	*N* = 332	%	
Mode of delivery	CS	89	47.6	98	52.4	187	56.33	7.5
	SVD	78	53.8	67	46.2	145	43.67	8.1

Gestational age (weeks)	28–32	7	6.9	94	93.1	101	30.4	11.4
	33–37	160	69.3	71	30.7	231	69.6	10.5

Sepsis	No	74	53.6	64	43.4	138	41.57	8.3
	Yes	93	47.9	101	52.1	194	58.43	7.4

Respiratory distress syndrome	No	132	52.8	118	47.2	250	75.3	8.1
	Yes	35	42.7	47	57.3	82	24.7	6.7

Necrotizing enterocolitis	No	143	53.6	124	46.4	267	80.42	8.2
	Yes	24	36.9	41	63.1	65	19.58	5.8

Patent ductus arteriosus	No	160	51.1	153	49.9	313	94.28	7.9
	Yes	7	36.8	12	63.2	19	5.72	5.9

SGA	No	156	53.1	138	46.9	294	88.55	8.2
	Yes	11	28.9	27	71.1	38	11.45	4.4

APGAR score in the 5 minutes	≤5	64	43.2	84	56.8	148	44.58	6.9
	>5	103	56.0	81	44.0	184	55.42	8.5

Continuous positive airway pressure	No	105	61.8	65	38.2	170	51.2	9.5
	Yes	62	38.3	100	61.7	162	48.8	5.9

Kangaroo mother care	No	59	29.8	139	70.2	198	59.64	4.6
	Yes	108	80.6	26	19.4	134	40.36	12.3

**Table 3:** Baseline enteral feeding practice information of time to reach FEF among VLBW neonates at FHCSH, Bahir Dar City, northwest Ethiopia, from 2018 to 2021.

Variables		Outcome status				Total *N* = 332		IDR/100/PDO
		FEF	%	Censored	%	Frequency	%	
Feeding type	FM	59	46.5	68	53.5	127	38.25	7.3
	BM	108	52.7	97	47.3	205	61.75	8.1

The starting time of EF	>3 days	30	23.8	96	76.2	126	37.95	3.8
	≤3 days	137	66.5	69	33.5	206	62.05	10.1

Daily VA of feeding (kg/day)	10-25 mL	93	46	109	54	202	60.84	7.2
	30-40 mL	74	56.9	56	43.1	130	39.16	8.7

Feeding frequency	Q6 h	78	55.3	78	46.7	141	42.47	8.8
	Q2 h	38	40.9	55	59.1	93	28.01	6.3
	Q3 h	51	52.0	47	48	98	29.52	7.9

Prefeed residual aspiration	No	123	66.1	63	33.9	186	56.02	10
	Yes	44	30.1	102	69.9	146	43.98	4.7

*Note.* EF = enteral feeding; *Q* = every; mL = milliliter; FM = formula milk; BM = breast milk; FEF = full enteral feeding; VA = volume advancement; IDR = incidence density rate.

**Table 4:** Survival probabilities of FEF among VLBW neonates admitted at FHCSH, Bahir Dar City, northwest Ethiopia, from 2018 to 2021.

Time interval	Beginning total	FEF	Censored	Cumulative survival probability	95% CI
2-3	332	0	2	1	—
3-4	330	1	5	0.9969	0.9785–0.9996
4-5	324	4	10	0.9844	0.9630–0.9935
5-6	310	12	25	0.9447	0.9126–0.9653
6-7	273	27	15	0.8487	0.8019–0.8852
7-8	231	123	108	0.2589	0.2021–0.3192

*Note*. FEF = full enteral feeding.

**Table 5:** Scaled Schoenfeld residuals proportional hazard assumption test for each variable and overall global test among very low birth weight neonates at FHCSH, Bahir Dar City, northwest Ethiopia, from 2018 to 2021.

Predicators	Rho	Chi^2^	df	*p* value
Preeclampsia	0.03890	0.26	1	0.6101
Pregnancy-induced hypertension	0.04911	0.42	1	0.5162
Small for gestational age	0.02716	0.13	1	0.7177
Continuous positive airway pressure	0.07933	1.13	1	0.2881
Kangaroo mother care	−0.00903	0.01	1	0.9051
Residual aspiration	0.04611	0.37	1	0.5435
Gestational age	0.11216	2.28	1	0.1313
The starting time of EF	0.09311	1.61	1	0.2043
Global test		5.64	8	0.6875

**Table 6:** Multivariable Cox regression analysis predictors of time to reach FEF among VLBW neonates who were admitted at FHCSH, Bahir Dar City, northwest Ethiopia, from 2018 to 2021 (*N* = 332).

Variables	Category	Event	Censored	CHR (95% CI)	AHR (95% CI)
Preeclampsia	No	157	142	1.7 (0.90–3.23)	1.19 (0.65–2.28)
	Yes	10	23	1	1

Pregnancy-induced hypertension	No	156	112	3.1 (1.68–5.71)	2.1 (1.12–3.94)^*∗*^
	Yes	11	53	1	1

Gestational age	28–32	7	94	1	1
	33–37	160	71	7.8 (3.67–16.67)	5 (2.29–11.13)^*∗∗*^

Starting time of trophic feeding	>3 days of age	30	96	1	1
	≤3 days of age	137	69	2.3 (1.58–3.48)	1.56 (1.03–2.35)^*∗*^

SGA	No	156	138	1.8 (1.02–3.47)	1.78 (0.95–3.33)
	Yes	11	27	1	1

CPAP	No	105	65	1.6 (1.17–2.19)	1.20 (0.87–1.65)
	Yes	62	100	1	1

KMC practice	No	59	139	1	1
	Yes	108	26	2.6 (1.91–3.61)	1.42 (1.01–2.00)^*∗*^

Prefeed residual aspiration	No	123	63	1.9 (1.36–2.72)	1.42 (1.002–2.03)^*∗*^
	Yes	44	102		

*Note.* ^*∗*^ *p* value <0.05, ^*∗∗*^*p* value ≤0.001, both ^*∗*^ and ^*∗∗*^ indicate statistically significant variables in the multivariable analysis. SGA = small for gestational age; CPAP = continuous positive airway pressure.

## Data Availability

The data used in this manuscript are available upon reasonable request by contacting one of the corresponding authors via e-mail.

## Abbreviations

AGA: Appropriate for gestational age
APGAR: Appearance, Pulse, Grimace, Activity, and Respiration
BSc: Bachelor's Science
CI: Confidence interval
CPAP: Continuous positive airway pressure
CS: Cesarian section
FMOH: Federal Ministry of Health
HR: Hazards ratio
ICU: Intensive care unit
IDR: Incidence density rate
INO_2_: Intranasal oxygen
IQR: Interquartile range
KMC: Kangaroo mother care
MM: Mother's milk
NEC: Necrotizing enterocolitis
NICU: Neonatal intensive care unit
NIMV: Noninvasive mechanical ventilator
PCHN: Pediatric and Child Health Nursing
PDA: Patent ductus arteriosus
PN: Parenteral nutrition
PPROM: Premature prolonged rupture of membrane
RDS: Respiratory distress syndrome
SGA: Small for gestational age
TFEF: Time to full enteral feeding
VD: Vaginal delivery
VLBW: Very low birth weight
WHO: World Health Organization.
